# Sugammadex and urinary retention after hysterectomy: A propensity-matched cohort study

**DOI:** 10.17305/bb.2023.9569

**Published:** 2024-04-01

**Authors:** Mariana L De Lima Laporta Miranda, Michelle A Ochs Kinney, Jamie N Bakkum-Gamez, Darrell R Schroeder, Juraj Sprung, Toby N Weingarten

**Affiliations:** 1 Department of Anesthesiology and Perioperative Medicine, Mayo Clinic School of Graduate Medical Education, Mayo Clinic College of Medicine and Science, Rochester, MN, USA; 2 Department of Obstetrics and Gynecology, Mayo Clinic, Rochester, MN, USA; 3 Division of Clinical Trials and Biostatistics, Mayo Clinic, Rochester, MN, USA

**Keywords:** General anesthesia, glycopyrrolate, neuromuscular blocking drugs, sugammadex, urinary retention

## Abstract

Postoperative urinary retention (POUR) is a well-known complication after gynecologic surgery. Our objective was to investigate whether the choice of a pharmacologic agent for reversing neuromuscular blockade at the end of a hysterectomy is a risk factor for POUR. Among adult patients undergoing hysterectomy with general anesthesia from 2012 to 2017, those who received aminosteroid nondepolarizing neuromuscular agents followed by pharmacologic reversal were identified, and electronic health records were reviewed. The cohort was dichotomized into two groups by the reversal agent: 1) sugammadex and 2) neostigmine with glycopyrrolate. The primary outcome, POUR, was defined as unplanned postoperative bladder recatheterization. A propensity-adjusted analysis was performed to investigate the association between POUR and reversal agent by using inverse probability of treatment weighting to adjust for potential confounders. We identified 1974 patients, of whom 1586 (80.3%) received neostigmine-glycopyrrolate and 388 (19.7%) received sugammadex for reversal of neuromuscular blockade. The frequency of POUR was 24.8% (393/1586) after reversal with neostigmine-glycopyrrolate and 18.3% (71/388) with sugammadex. Results from the propensity-adjusted analysis showed that sugammadex was associated with a lower POUR risk than neostigmine-glycopyrrolate [odds ratio (OR) 0.53, 95% confidence interval (CI) 0.37–0.76, *P* < 0.001]. A post hoc analysis of sugammadex recipients who received glycopyrrolate for another indication showed a higher POUR risk than among those who did not receive glycopyrrolate (OR 1.86, 95% CI 1.07–3.22, *P* ═ 0.03). The use of sugammadex to reverse aminosteroid neuromuscular blocking agents is associated with the decreased risk of POUR after hysterectomy. A potential mechanism is the omission of glycopyrrolate, which is coadministered with neostigmine to mitigate unwanted cholinergic effects.

## Introduction

Postoperative urinary retention (POUR) complicates 20%–25% of gynecologic surgical procedures [1–3] because of factors, such as surgical manipulation and general anesthesia [4]. Known risk factors for POUR include older age, intraoperative fluid volume, lengthy procedures, neuraxial anesthesia, and prior pelvic surgery [4–6].

Neuromuscular blocking agents (NMBAs) require pharmacologic reversal to mitigate the risk of postoperative respiratory complications. Traditionally, reversal of NMBAs is achieved with a cholinesterase inhibitor (e.g., neostigmine). However, due to unwanted muscarinic effects, the cholinesterase inhibitor is coadministered with an anticholinergic agent (e.g., glycopyrrolate). Sugammadex, a novel neuromuscular blockade-reversal agent that is devoid of muscarinic activity, does not require anticholinergic coadministration. In the past two years, studies of patients undergoing inguinal herniorrhaphy, video-assisted thoracic surgery, cholecystectomy, and ambulatory surgery have shown a lower risk of POUR with sugammadex for NMBA reversal than with traditional reversal agents [7–10]. The absence of an anticholinergic agent during reversal with sugammadex is the hypothesized mechanism for the observed difference [8, 9].

Previous investigations of POUR after gynecologic procedures do not account for the neuromuscular blockade-reversal agent [2, 4, 11, 12]. However, a reduction of POUR frequency after reversal with sugammadex cannot be assumed for gynecologic procedures because of direct surgical instrumentation of the genitourinary tract. We, therefore, retrospectively analyzed a large cohort of patients undergoing hysterectomy to determine whether an association could be identified between the reversal agent and the frequency of POUR. We hypothesized that using sugammadex would be associated with a lower rate of POUR than using traditional reversal agents.

## Materials and methods

### Study design

The aim of this retrospective cohort study was to assess whether the rates of POUR after hysterectomy differ by the agent used to reverse neuromuscular blockade.

### Clinical practice

The study was performed at a high-volume surgical practice in a large quaternary-care academic institution. A supervising anesthesiologist directed anesthetic care provided by an anesthesia resident, student nurse anesthetist, or certified registered nurse anesthetist. Muscle relaxation is typically achieved with an aminosteroid NMBA (vecuronium or rocuronium), which is reversed with neostigmine and glycopyrrolate (hereafter neostigmine-glycopyrrolate) or with sugammadex at the discretion of the attending anesthesiologist. Sugammadex was introduced into clinical practice at our institution on October 1, 2016. During the hysterectomy, the patient’s urethra is catheterized after anesthesia is induced. Urinary catheters were removed 6 h after surgery and then patients underwent a passive voiding trial. After the first postoperative void, the bladder was scanned, and if the total volume of urine was less than 50% of prevoid volume or less than 150 mL no further assessments were required. If the patient was unable to void after 6 h of catheter removal, then the surgical service was notified. Generally, patients who failed these metrics underwent recatheterization, but this decision was ultimately made by the surgical team.

### Patient selection

The electronic health records were reviewed for consecutive adult patients who underwent hysterectomy with general anesthesia from January 1, 2012, to December 31, 2017. This cohort was previously used to investigate respiratory depression after hysterectomy and was described in detail [13]. Participants were excluded if they were not administered aminosteroid NMBAs or a neuromuscular blockade-reversal agent, or if they underwent combined obstetric procedures, extensive surgical resection (e.g., pelvic exenteration, liver resection, nephrectomy, bowel resection, or omentectomy), or emergency surgery.

### Data abstraction

The following data were abstracted from the health records: demographic characteristics, potentially relevant comorbid conditions, and perioperative and postoperative data. Records of the perioperative course were reviewed for surgical approach (vaginal, robotic-assisted, laparotomy, and concurrent vaginal repair), surgical duration, intravenous fluids, perioperative medications with opioid dose converted to oral morphine equivalents, and agent for reversing neuromuscular blockade. The postoperative course was evaluated for the presence of the primary outcome, POUR, defined as the inability to void postoperatively requiring unplanned new bladder catheterization, with either placement of an indwelling catheter or intermittent catheterization within the first 24 postoperative h. Other postoperative data collected were hospital length of stay and the incidence within the first 24 postoperative h of the following: nausea and vomiting (defined as administration of rescue antiemetics), severe pain (defined as a pain score ≥ 7 on a 10-point pain scale where 0 indicates no pain and 10 indicates the worst pain imaginable), and opioid analgesic use. The presence of urinary tract infections within the first seven postoperative days and an emergency department visit within the first 48 h after hospital discharge for possible urinary retention were also evaluated.

### Ethical statement

This study was approved by our institutional review board (protocol No. 19-002835, approved on July 25, 2019). Consistent with Minnesota Statute 144.295, all participants enrolled in the study had authorized use of their health records for retrospective research.

### Statistical analysis

POUR was the primary outcome of interest. To investigate the association of sugammadex with POUR frequency and to reduce potential treatment selection bias or confounding effects, significant clinical and procedural differences and adjusted by using inverse probability of treatment weighting (IPTW). Patient-specific estimates for the probability of receiving sugammadex were obtained by using logistic regression analysis, with sugammadex as the dependent variable and with all patient and procedural characteristics listed in Table 1 as explanatory variables. These variables were selected a priori and were selected because they could potentially influence the rates of POUR. The standardized (mean) difference was calculated for each characteristic before and after propensity score adjustment to evaluate the balance across groups. POUR was analyzed by using logistic regression with robust variance estimates. Patient and procedural characteristics are summarized for each reversal-agent group (sugammadex or neostigmine-glycopyrrolate) with a mean (SD) for continuous variables and count and percentage for categorical variables. Findings from the regression analysis are summarized by presenting the odds ratio (OR) and corresponding 95% confidence interval (CI), where an OR less than 1 corresponded to a lower likelihood of POUR for patients receiving sugammadex compared with neostigmine-glycopyrrolate. Hospital length of stay was treated as an ordinal variable and compared between groups by using a proportional odds model.

Some patients in the sugammadex group also received glycopyrrolate for bradycardia treatment. A post hoc logistic regression analysis of these patients was performed to assess whether POUR frequency was associated with the additional use of glycopyrrolate. This analysis was restricted to patients treated with sugammadex and included covariates for age, type of surgery, repair, duration of surgery, intraoperative crystalloids, and intraoperative opioids. An additional post hoc logistic regression analysis was performed to determine if the frequency of POUR was associated with the dose of glycopyrrolate. In all cases, two-tailed *P* values < 0.05 were considered significant. Analyses were performed with SAS version 9.4 software (SAS Institute Inc.).

## Results

Overall, 1974 patients underwent hysterectomy under general anesthesia during the study period and were included in the study. In total, 1586 patients (80.3%) were in the neostigmine-glycopyrrolate group (i.e., received neostigmine-glycopyrrolate for reversal of neuromuscular blockade), and 388 (19.7%) were in the sugammadex group. The median dose (IQR) was 4 (3–5) mg of neostigmine with 0.6 (0.4–0.8) mg of glycopyrrolate, and 160 (140–200) mg of sugammadex. Among the included procedures, 587 (29.7%) were performed with an abdominal approach, 851 (43.1%) were robotic-assisted, and 536 (27.2%) were performed via the vaginal approach (Table 1). The standardized difference was greater than 0.20 for age, body mass index, scopolamine, dexamethasone, crystalloids, and intraoperative opioids without propensity score adjustment, which indicated a meaningful imbalance between groups. After IPTW adjustment, the groups were well balanced (Table 1). The administration of opioids during anesthesia recovery was not included in the propensity scores for the IPTW. Opioid analgesics were administered to 1023 (64.5%) of the neostigmine-glycopyrrolate and 244 (62.9%) of the sugammadex patients (*P* ═ 0.552) and of those who received an opioid, the median dose was 23 [12, 43] mg OME for the neostigmine-glycopyrrolate and 20 [10, 35] mg OME for the sugammadex patients (*P* < 0.001).

**Table 1:** Standardized difference of covariates after the inverse probability of treatment weighting of sample*

**Variable**	**Sugammadex (*n* ═ 388)**	**Neostigmine-glycopyrrolate (*n* ═ 1586)**	**Standardized difference**	
			**Unadjusted**	**IPTW**
Age, mean (SD), years	57.3 (13.1)	56.2 (13.3)	0.082	0.041
Body mass index^**^, mean (SD)	30.1 (7.2)	32.0 (9.5)	0.228	0.107
Diabetes	41 (10.6)	187 (11.8)	0.039	0.012
Hypertension	161 (41.5)	656 (41.4)	0.003	0.044
Kidney disease	0 (0)	0 (0)		
Home benzodiazepine use	29 (7.5)	106 (6.7)	0.031	0.025
Home opioid use	45 (11.6)	238 (15.0)	0.100	0.045
*Procedure type*				
Abdominal	96 (24.7)	491 (31.0)	0.139	0.141
Robotic-assisted	181 (46.6)	670 (42.2)	0.089	0.076
Vaginal	111 (28.6)	425 (26.8)	0.040	0.065
Vaginal repair	49 (12.6)	187 (11.8)	0.026	0.002
Scopolamine	65 (16.8)	132 (8.3)	0.257	0.002
Intraoperative dexamethasone	351 (90.5)	1285 (81.0)	0.273	0.043
Intraoperative crystalloids, mean (SD), mL	2952 (1254)	2698 (1200)	0.207	0.012
Intraoperative opioids, mean (SD), mL OME	88.9 (35.9)	99.5 (38.5)	0.283	0.157
Duration of surgery, mean (SD), min	180.2 (87.8)	172.1 (81.8)	0.095	0.184

^*^Data are presented as No. (%) except as otherwise indicated. ^**^Calculated as weight in kilograms divided by height in meters squared. OME: Oral morphine equivalents; SD: Standard deviation; IPTW: Inverse probability of treatment weighting.

The frequency of POUR was 24.8% (393/1586) for the neostigmine-glycopyrrolate group and 18.3% (71/388) for the sugammadex group (unadjusted OR 0.68, 95% CI 0.51–0.90, *P* ═ 0.007, IPTW-adjusted OR 0.53, 95% CI 0.37–0.76, *P* < 0.001). The median (IQR) time from the end of surgery to diagnosis of POUR was 10.1 (5.3–22.9) h. In the sugammadex group, 128 patients (32.9%) received glycopyrrolate (median [IQR] dose 0.2 [0.2–0.2] mg) to treat bradycardia intraoperatively. Results from the post hoc analysis showed the frequency of POUR to be higher among patients who also received glycopyrrolate than among those who did not (25.0% vs 15.0%, unadjusted OR 1.89, 95% CI 1.12–3.19, *P* ═ 0.02, covariate-adjusted OR 1.86, 95% CI 1.07–3.22, *P* ═ 0.03).

To further explore if the dose of glycopyrrolate was associated with POUR risk, an additional logistic regression analysis restricted to the neostigmine-glycopyrrolate group was performed and found the likelihood of POUR increased with higher doses of glycopyrrolate, OR 1.04, 95% CI 1.01–1.07, per 0.1-mg glycopyrrolate dose, *P* ═ 0.034. A similar analysis was not conducted for the sugammadex patients because there was insufficient variability of glycopyrrolate dose in this group.

The postoperative course was similar between patients in whom POUR did or did not develop, however, patients with POUR had higher rates of severe pain (*P* ═ 0.03) and postoperative nausea and vomiting (*P* < 0.001), and had a more extended hospitalization in the subgroups of robotic-assisted hysterectomy and abdominal hysterectomy (Table 2).

**Table 2:** Postoperative outcomes of patients with or without postoperative urinary retention after hysterectomy*

**Outcome**	**Overall**			**Vaginal**			**Robotic-assisted**			**Abdominal**		
	**POUR (*n* ═ 464)**	**No POUR (*n* ═ 1510)**	***P* value**	**POUR (*n* ═ 112)**	**No POUR (*n* ═ 424)**	***P* value**	**POUR (*n* ═ 242)**	**No POUR (*n* ═ 609)**	***P* value**	**POUR (*n* ═ 110)**	**No POUR (*n* ═ 477)**	***P* value**
Length of stay, median (IQR), d	1.4 (1.2 – 2.3)	1.4 (1.2 – 2.3)	0.70	1.2 (1.0 – 1.4)	1.2 (1.1 – 1.5)	0.41	1.2 (1.1 – 1.4)	1.1 (1.0 – 1.2)	<0.001	2.6 (2.2 – 4.3)	2.2 (2.2 – 3.3)	0.04
Next-day discharge	305 (65.7)	1,008 (66.8)	0.69	91 (81.3)	353 (83.3)	0.67	195 (80.6)	548 (90.0)	<0.001	19 (17.3)	107 (22.4)	0.25
Discharge > 3 d	77 (16.6)	210 (13.9)	0.15	9 (8.0)	13 (3.1)	0.03	16 (6.6)	20 (3.3)	0.04	52 (47.3)	177 (37.1)	0.05
PONV	160 (34.5)	386 (25.6)	<0.001	36 (32.1)	87 (20.7)	0.01	61 (25.2)	81 (13.3)	<0.001	63 (57.3)	218 (45.7)	0.03
Severe pain^**^	196 (42.2)	549 (36.4)	0.03	56 (50.0)	176 (41.9)	0.13	76 (31.4)	142 (23.3)	0.02	64 (58.2)	231 (48.5)	0.07
Opioids, median (IQR), mg OME^***^	13.6 (3.4 – 30.3)	12.7 (0 – 30.2)	0.39	19.6 (4.5 – 37.7)	16.2 (3.9 – 35.7)	0.43	9.6 (0 – 22.4)	6.8 (0 - 19.7)	0.08	19.4 (10.2 – 37.4)	18.6 (6.6 – 38.3)	0.40
Urinary tract infection	22 (4.7)	54 (3.6)	0.27	7 (6.3)	19 (4.5)	0.46	9 (3.7)	18 (3.0)	0.67	6 (5.5)	17 (3.6)	0.41
Emergency department^****^	19 (4.1)	46 (3.0)	0.30	4 (3.6)	11 (2.6)	0.53	6 (2.5)	17 (2.8)	>0.99	9 (8.2)	18 (3.8)	0.07

^*^Data are presented as No. (%) unless otherwise indicated. ^**^Severe pain is defined as pain ≥ 7 on a 0–10 numeric pain scale where 0 indicates no pain and 10 indicates the worst pain imaginable. ^***^Opioids for 24 h postoperatively. ^****^Emergency department visit within 48 h of hospital discharge for possible urinary retention. OME: Oral morphine equivalents; POUR: Postoperative urinary retention; PONV: Postoperative nausea and vomiting.

## Discussion

The main observation of this study was that the use of sugammadex for reversing NMBAs after hysterectomy was associated with a 47.0% lower risk of POUR compared with traditional reversal with neostigmine-glycopyrrolate. Of interest, subgroup analysis of patients in the sugammadex group who were coadministered glycopyrrolate for another indication (bradycardia) showed they had higher rates of POUR than patients who received sugammadex without perioperative glycopyrrolate administration. Outcomes of patients with POUR were worse for higher rates of postoperative nausea and vomiting and pain.

The lower POUR risk observed in the sugammadex group after hysterectomy is consistent with findings from several reports examining the type of neuromuscular blockade-reversal agent and association with POUR after other surgical procedures. Among 181 patients undergoing laparoscopic inguinal herniorrhaphy, the use of sugammadex was associated with more than 80% reduction in POUR frequency compared with the use of neostigmine-glycopyrrolate (2/75 [3%] vs 16/106 [15%], respectively) [8]. After video-assisted thorascopic procedures for 276 patients, the POUR incidence was 14% with neostigmine-glycopyrrolate as the reversal agent vs 4% with sugammadex [9]. In a prospective study of 75 patients undergoing laparoscopic cholecystectomy, urinary retention was reported for 15.8% of patients who had reversal with neostigmine-glycopyrrolate compared with 3% of patients who received sugammadex [7]. Another small prospective study of 37 patients undergoing ambulatory surgery showed the rate of POUR to be 10.5% after reversal with neostigmine-glycopyrrolate vs 0% after reversal with sugammadex [10]. In the current study, the incidence of POUR was greater, and the magnitude of POUR risk reduction was less, in the sugammadex group than that reported in previous related studies [7–9]. We speculate that these differences arise because hysterectomy directly affects the genitourinary system, which inherently increases the risk of POUR. Indeed, the rates of POUR in our study are similar to those in other reports of POUR after gynecologic surgery [1–3].

Although the mechanism is unknown for the different POUR rates between the two reversal agents, we speculate that coadministering an anticholinergic agent when a cholinesterase inhibitor is administered has a central role. Acetylcholine causes contraction of the bladder detrusor muscle leading to micturition [14]. Anticholinergic agents inhibit acetylcholine activity, and urinary retention is a known adverse effect of these medications. For example, results from a study of ambulatory urogynecologic procedures showed that patients with high anticholinergic exposure had an increased risk of a failed result of a postoperative void trial [15]. Our post hoc result that POUR rates were increased among patients who received both sugammadex and glycopyrrolate supports the theory that the anticholinergic is the agent responsible for higher POUR rates after reversal with neostigmine-glycopyrrolate.

Recent guidelines from the European Society of Anaesthesiology and Intensive Care [16] and the American Society of Anesthesiologists [17] advocate a more conservative practice when utilizing neostigmine to reverse non-depolarizing muscle relaxants with both lower dose and more stringent indication. These practice changes would also allow for lower doses of glycopyrrolate coadministration. The timeframe of this study predates these new guidelines. Our post hoc analysis of the dose of glycopyrrolate and the development of POUR found an increase in risk with higher doses of glycopyrrolate among the neostigmine group. We could not perform this analysis in the sugammadex group because most patients received a 0.2 mg dose to treat bradycardia and there was insufficient variability to assess dose response. These results support our hypothesis that the anticholinergic effects of glycopyrrolate in a neostigmine-glycopyrrolate reversal are the mechanism for higher rates of POUR in this group. However, our results and results from other retrospective studies merit confirmation with a prospective trial, and such a trial should incorporate contemporary practice with the updated neostigmine practice as this has the potential to yield different results.

This study has the limitations inherent in a retrospective design. Importantly, because sugammadex was introduced into clinical practice in 2016, there is the possibility that unaccounted clinical improvements over time may have biased results. However, there were no substantial practice changes made to catheter management during the study timeframe. Because the surgical service made the ultimate decision regarding recatheterization, there is the potential for unaccounted practice differences introducing an element of bias. Therefore, the results should be regarded as hypothesis generating. Still, these findings suggest the need to perform future randomized clinical trials comparing POUR rates between patients receiving sugammadex and those receiving neostigmine-glycopyrrolate.

## Conclusion

The rate of POUR in this large cohort of patients undergoing hysterectomy was lower among patients whose neuromuscular blockade was reversed with sugammadex compared with neostigmine-glycopyrrolate. This finding could be due to avoiding the anticholinergic glycopyrrolate.

## References

[1] Ghezzi F, Cromi A, Uccella S, Colombo G, Salvatore S, Tomera S (2007 Nov-Dec). Immediate Foley removal after laparoscopic and vaginal hysterectomy: determinants of postoperative urinary retention. J Minim Invasive Gynecol.

[2] Yune JJ, Cheng JW, Wagner H, Kim J, Hardesty JS, Siddighi S (2018 Jun). Postoperative urinary retention after pelvic organ prolapse repair: vaginal versus robotic transabdominal approach. Neurourol Urodyn.

[3] Behbehani S, Delara R, Yi J, Kunze K, Suarez-Salvador E, Wasson M (2020 Mar-Apr). Predictors of postoperative urinary retention in outpatient minimally invasive hysterectomy. J Minim Invasive Gynecol.

[4] Baldini G, Bagry H, Aprikian A, Carli F (2009 May). Postoperative urinary retention: anesthetic and perioperative considerations. Anesthesiology.

[5] Dreijer B, Moller MH, Bartholdy J (2011 Mar). Post-operative urinary retention in a general surgical population. Eur J Anaesthesiol.

[6] Hansen BS, Soreide E, Warland AM, Nilsen OB (2011 May). Risk factors of post-operative urinary retention in hospitalised patients. Acta Anaesthesiol Scand.

[7] Han J, Oh AY, Jeon YT, Koo BW, Kim BY, Kim D (2021 Mar 1). Quality of recovery after laparoscopic cholecystectomy following neuromuscular blockade reversal with neostigmine or sugammadex: a prospective, randomized, controlled trial. J Clin Med.

[8] Valencia Morales DJ, Stewart BR, Heller SF, Sprung J, Schroeder DR, Ghanem OM (2021 May 24). urinary retention following inguinal herniorrhaphy: role of neuromuscular blockade reversal. Surg Laparosc Endosc Percutan Tech.

[9] Daquioag TK, Mele NJ, Peterson DR, Steinhorst JD, Schultz SJ, Steege JR (2022 Jan). Urinary retention after video-assisted thoracoscopic surgery: role of neuromuscular blockade reversal. J Cardiothorac Vasc Anesth.

[10] Fiorda Diaz J, Echeverria-Villalobos M, Esparza Gutierrez A, Dada O, Stoicea N, Ackermann W (2022). Sugammadex versus neostigmine for neuromuscular blockade reversal in outpatient surgeries: a randomized controlled trial to evaluate efficacy and associated healthcare cost in an academic center. Front Med (Lausanne).

[11] Kandadai P, Saini J, Patterson D, O’Dell K, Flynn M (2015 Sep-Oct). Urinary retention after hysterectomy and postoperative analgesic use. Female Pelvic Med Reconstr Surg.

[12] Sappenfield EC, Scutari T, O’Sullivan DM, Tulikangas PK (2021 Mar). Predictors of delayed postoperative urinary retention after female pelvic reconstructive surgery. Int Urogynecol J.

[13] Laporta ML, Kinney MO, Schroeder DR, Sprung J, Weingarten TN (2021 Jun 1). Postoperative respiratory depression after hysterectomy. Bosn J Basic Med Sci.

[14] Rubinstein R, Nissenkorn I, Cohen S (1986 Apr 9). A study of the contractile response to acetylcholine in human ileal and detrusor muscle: origin of the low efficacy of acetylcholine. Eur J Pharmacol.

[15] Walter PJ, Dieter AA, Siddiqui NY, Weidner AC, Wu JM (2014 May-Jun). Perioperative anticholinergic medications and risk of catheterization after urogynecologic surgery. Female Pelvic Med Reconstr Surg.

[16] Fuchs-Buder T, Romero CS, Lewald H, Lamperti M, Afshari A, Hristovska A (2023 Feb). Peri-operative management of neuromuscular blockade: a guideline from the European Society of Anaesthesiology and Intensive Care. Eur J Anaesthesiol.

[17] Thilen SR, Weigel WA, Todd MM, Dutton RP, Lien CA, Grant SA (2023 Jan). 2023 American Society of Anesthesiologists practice guidelines for monitoring and antagonism of neuromuscular blockade: a report by the American Society of Anesthesiologists task force on neuromuscular blockade. Anesthesiology.

